# RNA-seq Sample Preparation Kits Strongly Affect Transcriptome Profiles of a Gas-Fermenting Bacterium

**DOI:** 10.1128/spectrum.02303-22

**Published:** 2022-07-27

**Authors:** Lorena Azevedo de Lima, Kristina Reinmets, Lars Keld Nielsen, Esteban Marcellin, Audrey Harris, Michael Köpke, Kaspar Valgepea

**Affiliations:** a ERA Chair in Gas Fermentation Technologies, Institute of Technology, University of Tartu, Tartu, Estonia; b Australian Institute for Bioengineering and Nanotechnology, The University of Queensland, St. Lucia, Australia; c ARC Centre of Excellence in Synthetic Biology, The University of Queensland, St. Lucia, Australia; d Novo Nordisk Foundation Center for Biosustainability, Technical University of Denmark, Kongens Lyngby, Denmark; e LanzaTech Inc., Skokie, Illinois, USA; University of Exeter

**Keywords:** acetogen, RNA sequencing, RNA-seq, transcriptome profiling, transcriptomics

## Abstract

Transcriptome analysis via RNA sequencing (RNA-seq) has become a standard technique employed across various biological fields of study. The rapid adoption of the RNA-seq approach has been mediated, in part, by the development of different commercial RNA-seq library preparation kits compatible with standard next-generation sequencing (NGS) platforms. Generally, the essential steps of library preparation, such as rRNA depletion and first-strand cDNA synthesis, are tailored to a specific group of organisms (e.g., eukaryotes versus prokaryotes) or genomic GC content. Therefore, the selection of appropriate commercial products is of crucial importance to capture the transcriptome of interest as closely to the native state as possible without introduction of technical bias. However, researchers rarely have the resources and time to test various commercial RNA-seq kits for their samples. This work reports a side-by-side comparison of RNA-seq data from *Clostridium autoethanogenum* obtained using three commercial rRNA removal and strand-specific library construction products of NuGEN Technologies, Qiagen, and Zymo Research and assesses their performance relative to published data. While all three vendors advertise their products as suitable for prokaryotes, we found significant differences in their performance regarding rRNA removal, strand specificity, and most importantly, transcript abundance distribution profiles. Notably, RNA-seq data obtained with Qiagen products were most similar to published data and delivered the best results in terms of library strandedness and transcript abundance distribution range. Our results highlight the importance of finding appropriate organism-specific workflows and library preparation products for RNA-seq studies.

**IMPORTANCE** RNA-seq is a powerful technique for transcriptome profiling while involving elaborate sample processing before library sequencing. We show that RNA-seq library preparation kits can strongly affect the outcome of an RNA-seq experiment. Although library preparation benefits from the availability of various commercial kits, choosing appropriate products for the specific samples can be challenging for new users or for users working with unconventional organisms. Evaluating the performance of different commercial products requires significant financial and time investments infeasible for most researchers. Therefore, users are often guided in their choice of kits by published data involving similar input samples. We conclude that important consideration should be given to selecting sample processing workflows for any given organism.

## INTRODUCTION

Gene transcription is a fundamental process that mediates a vast number of intracellular and environmental responses in every cell. Therefore, understanding transcriptional states of any organism of choice can shed light on basic biological processes as well as ways to direct and control cellular behavior. Insights into cellular transcriptional profiles or transcriptomes (i.e., a complete set of transcripts in a cell and their quantities) have vastly expanded in the last decade due to rapid development and high accessibility of next-generation sequencing (NGS) platforms (1). Meanwhile, constant improvement of commercial library construction products has greatly contributed to the rapid adaptation and evolution of RNA sequencing applications, for example, RNA sequencing (RNA-seq) (2), nascent RNA sequencing (3), Ribo-seq (4), and differential RNA-seq (5). RNA-seq is the most common application, as it allows both mapping and quantification of transcriptomes.

While RNA-seq has become widely used across all fields of biological sciences, obtaining high-quality data of the transcriptome under investigation nevertheless requires careful planning, extensive sample processing, and considerable resources. The availability of commercial RNA-seq library preparation kits tailored to a variety of organisms, experimental approaches, and sequencing platforms has made RNA-seq accessible even to nonexpert users. When planning to do an RNA-seq experiment for the first time, researchers often consult existing literature to see which sample preparation protocols and products have been previously used with their organism of interest. However, working with unconventional microorganisms that have not yet been extensively studied via RNA-seq can make it difficult to decide which commercial kits might be most suitable.

We previously achieved high-quality transcriptome profiling using RNA-seq for the gas-fermenting bacterium Clostridium autoethanogenum (6–8), an unconventional microbe that is also used as a cell factory in commercial-scale gas fermentation for the production of low-carbon fuels and chemicals from waste feedstocks (9). In addition to preparation of cDNA libraries before sequencing, removal of rRNA from the extracted total RNA is needed to ensure efficient transcriptome-wide mRNA detection and quantification as >80–90% of total cellular RNA is rRNA (10, 11). In our previous studies (6–8), we used Illumina kits for rRNA removal and library preparation, but when we set out to start a large-scale RNA-seq survey of the same organism in late 2019, the Illumina Ribo-Zero rRNA removal kit was discontinued, and we had to look for alternatives. However, selecting an efficient rRNA removal method for bacterial samples is nontrivial, as enrichment of non-rRNA transcripts based on polyadenylated RNA [poly(A)] selection used in most commercial kits (developed for eukaryotes) is not applicable for bacterial RNA due to the lack of poly(A) tails. One also has to ensure the compatibility of the rRNA removal and cDNA library preparation methods.

To make an informed decision on the best commercial products for RNA-seq library preparation for C. autoethanogenum, we aimed to test kits from three vendors that are advertised to ensure efficient rRNA removal and to be compatible with a variety of bacterial species and Illumina sequencing platforms. This work reports a side-by-side comparison of RNA-seq data obtained from the same *C. autoethanogenum* input samples using rRNA removal and strand-specific library construction kits from NuGEN Technologies, Qiagen, and Zymo Research and assesses their performance relative to published data. Transcriptome profiles revealed significant differences between the kits regarding rRNA removal efficiency, sequencing read strand specificity, and strikingly, transcript abundance distribution profiles. Our work shows that Qiagen kits yield the most reliable data of the three we tested and highlights the importance of appropriate sample preparation for RNA-seq analysis in bacteria.

## RESULTS AND DISCUSSION

### Experimental design.

**FIG 1:**
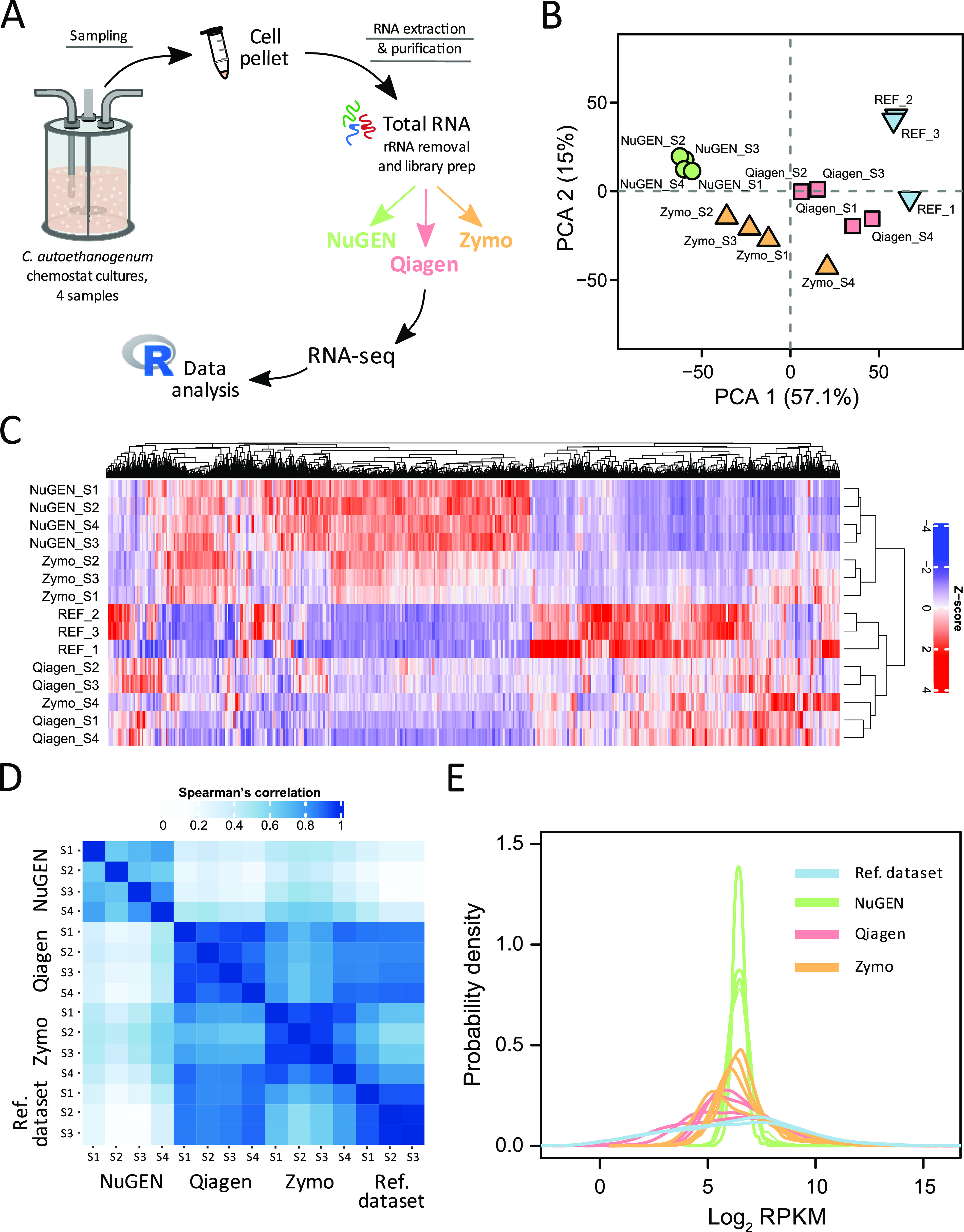
RNA-seq results were strongly affected by rRNA removal and library construction kits. (A) Experimental design of the work. (B) PCA of transcript abundances. (C) Hierarchical clustering of individual transcript abundances. (D) Spearman’s correlation analysis of transcript abundances. (E) Probability density plots of transcript abundances. The reference data set refers to high-BC samples in GEO accession number GSE90792. REF, reference data set. rRNA transcript abundances were removed prior to data analysis to avoid bias from varied efficiencies of rRNA removal between kits.

We evaluated the performance of three commercial rRNA removal and strand-specific library construction kits by NuGEN, Qiagen, and Zymo (see Materials and Methods for details) for RNA-seq analysis of C. autoethanogenum autotrophic cultures (Fig. 1A). To assess the ability of the selected commercial kits to capture the transcriptomic profile of *C. autoethanogenum* under various culture conditions, we used four samples, each obtained from one of four bioreactor continuous culture experiments grown on two different feed gas mixes (CO or CO + CO_2_ + H_2_ [syngas]) and dilution rates (i.e., specific growth rates of 1 or 2 day^−1^). Each sample represented a single biological replicate. Both feed gas composition (12) and specific growth rate (13) of the culture have profound effects on the culture phenotype (e.g., gas uptake, product distribution, metabolic fluxes). We extracted and prepared total RNA from the four samples using a previously established workflow optimized for *C. autoethanogenum* (6). Next, total RNA for each sample was split between the NuGEN, Qiagen, and Zymo kits for rRNA removal and strand-specific RNA-seq library construction according to the vendors’ instructions. Finally, the 12 samples (four cultures times three kits) were examined by paired-end 75-bp sequencing on an Illumina MiSeq platform, followed by RNA-seq data analysis using established pipelines (6, 13).

### General statistics of RNA-seq data.

An average of 4.5 million raw reads per sample were obtained from the sequencing runs that were mapped after trimming to the reference genome of *C. autoethanogenum* NC_022592.1 (14) with an overall high success rate (Table 1). Namely, 98%, 93%, and 99% of reads were mapped on average for NuGEN, Qiagen, and Zymo, respectively, which resulted in a minimum 50-fold coverage of the *C. autoethanogenum* genome across samples (Table 1). We detected very low read duplication levels (<0.5%), suggesting a low chance of technical bias introduced during sample preparation. Surprisingly, a significant difference in the percentage of mapped reads that were assigned to genomic features (i.e., feature counts) was observed between NuGEN and the other two kits: an average of only 55% for NuGEN with 84% and 79% for Qiagen and Zymo, respectively (Table 1). Notably, this can be explained by the difference between NuGEN and the other two kits in the percentage of reads that mapped to the expected strand, as Qiagen and Zymo showed high correct strandedness at ~91% (sense) and ~84% (antisense), respectively, compared to NuGEN’s very poor strand specificity at ~58% (sense) (Table 1). Significant false strandedness for NuGEN could arise from either a substantial flaw in the respective workflow or, according to NuGEN, from faulty reagents in their kits (personal communication).

**TABLE 1:** General statistics of RNA-seq results of the three tested kits for rRNA removal and library construction

Kit	Sample name	No. of raw reads	Reads mapped (RPKM)	Coverage (fold)	Feature counts/mapped counts	Strandedness		rRNA RPKM/total RPKM
						Sense	Antisense	
NuGEN	NuGEN_S1	4,268,524	98%	72	59%	64%	36%	7%
	NuGEN_S2	6,004,018	99%	103	47%	50%	50%	2%
	NuGEN_S3	3,615,864	99%	62	52%	56%	44%	4%
	NuGEN_S4	4,248,288	98%	72	63%	61%	39%	15%
Qiagen	Qiagen_S1	4,911,456	98%	81	87%	95%	5%	15%
	Qiagen_S2	3,559,522	95%	57	79%	84%	16%	9%
	Qiagen_S3	3,289,702	93%	50	82%	88%	12%	6%
	Qiagen_S4	4,954,218	88%	74	88%	96%	4%	17%
Zymo	Zymo_S1	4,355,956	99%	71	82%	13%	87%	0.8%
	Zymo_S2	4,840,762	99%	79	70%	26%	74%	0.6%
	Zymo_S3	5,691,744	99%	93	78%	18%	82%	0.6%
	Zymo_S4	4,651,118	99%	76	87%	7%	93%	0.8%

### Varied efficiency of rRNA removal.

We next quantified rRNA removal efficiencies from the RNA-seq data using the percentages of rRNA transcript abundance per total transcript abundance, expressed as reads per kilobase of transcript per million mapped reads (RPKM) (see Table S1 in the supplemental material). Again, stark differences between the kits were observed, confirming that rRNA depletion from bacterial samples is nontrivial (Table 1). The Zymo kit demonstrated superior efficiency for rRNA removal during library preparation, with an abundance of <1% of rRNA transcripts. NuGEN’s higher variability in rRNA removal efficiency across the samples (2 to 15%, average of 7%) suggested that their approach of non-rRNA enrichment or AnyDeplete technology may be sensitive to sample-specific factors. Removal of rRNA for the Qiagen kit was slightly less efficient (~11%) than for NuGEN but still acceptable to ensure high coverage of transcriptome-wide mRNA detection and quantification (Table 1).

### Kit-specific grouping of transcriptome profiles.

Upon observing the differences in general RNA-seq metrics (outlined in Table 1), we were curious if different kits could also lead to varied transcriptome profiles. Indeed, principal component analysis (PCA) of transcript abundances revealed clear sample grouping by kit and not by the origin of the input RNA (Fig. 1B). To assess which of the three tested kits produced the most reliable transcriptome profiles, we also included published data in the PCA that we previously obtained using the same workflows but with Illumina kits for similar *C. autoethanogenum* culture conditions (6), termed here as the reference data set (high-biomass concentration samples in GEO accession number GSE90792). Notably, Qiagen data were grouped the closest to this reference data set, with NuGEN transcriptome profiles separating most distinctively (Fig. 1B). These observations were confirmed by hierarchical clustering of individual transcript abundances, which showed grouping of samples based on the kits and not based on the origin of the input RNA (Fig. 1C). The NuGEN and Zymo kits had a distinctively different clustering pattern than the Qiagen kit and the reference data set.

Clustering results agreed with Spearman’s correlation analysis of transcript abundances between samples, which showed Qiagen data were most similar to the reference data set (ρ ~ 0.86) (Fig. 1D). Within the three kits tested here, Qiagen and Zymo data showed higher similarity (ρ ~ 0.77 across the same samples) compared to the lower correlations between NuGEN and Qiagen (ρ ~ 0.33) and NuGEN and Zymo (ρ ~ 0.47) data.

### Differences in transcript abundance distribution profiles.

The quality of the kits could also be assessed by their sensitivity to detect transcripts across a range of abundances (i.e., transcriptome coverage, or depth). Transcript levels in bacteria generally span over 4 orders of magnitude (15–17), including in *C. autoethanogenum* (6) and other gas-fermenting bacteria (18, 19). Again, Qiagen data resembled the reference data set the closest by both the transcript abundance distribution profiles and good sensitivity, with transcript levels spanning over 4 orders of magnitude (from ~2 to ~39,000 RPKM; ~1 to 15 log_2_ RPKM) (Fig. 1E). Strikingly, the NuGEN kit showed very narrow transcript abundance distributions that covered only ~2 orders of magnitude (from ~22 to ~1,800 RPKM; ~4 to ~11 log_2_ RPKM), while Zymo data were positioned between Qiagen and NuGEN. According to Zymo (personal communication), such condensed distribution profiles could be caused by higher sensitivity of the workflow toward the presence of genomic DNA in the input sample, which can artificially inflate mRNA reads and with a more prominent effect on low-abundance transcripts, thereby pushing the left tail of the distribution to the right. This would also be consistent with the poorer strandedness of the Zymo and NuGEN data (Table 1) that arose from genomic DNA-originating reads. Our sample preparation workflow that was previously optimized for *C. autoethanogenum* (6) efficiently removed DNA from total RNA samples down to ~13 ± 2 ng/μL (average ± standard deviation), making up ~4% of the RNA concentration. Thus, additional steps to deplete DNA to extremely low levels are potentially required for the Zymo and NuGEN workflows. NuGEN data could be additionally explained by biased synthesis and amplification of cDNA using selective primers, compared to the general use of random primers in RNA-seq workflows.

Our work is important, as researchers rarely have the resources and time to test various commercial RNA-seq kits, which are advertised as suitable for multiple organisms with different genomic GC content, for their samples. The ability to capture the spectrum of transcript abundances as closely to the true cellular state as possible is crucial to accurately address research questions investigated via RNA-seq. Our work shows that rRNA removal and library construction kits can strongly affect RNA-seq outcomes. This is highly relevant for anyone establishing an RNA-seq pipeline for an organism or for researchers puzzled by unexpected RNA-seq results. We conclude that, at least for *C. autoethanogenum* RNA-seq studies, the Illumina and Qiagen kits are most suitable, by providing high sensitivity across a wide range of transcript levels, superior strand specificity, and sufficient rRNA removal, ensuring high coverage of transcriptome-wide mRNA detection and quantification.

## MATERIALS AND METHODS

### Bacterial strain and cultivation conditions.

A derivate of *Clostridium autoethanogenum* DSM 10061 strain, namely DSM 23693, deposited in the German Collection of Microorganisms and Cell Cultures (DSMZ), was used in all experiments and stored as a glycerol stock at −80°C. Full details of the cultivation conditions are reported in previous work (13). Shortly, cells were grown autotrophically in bioreactor chemostat continuous cultures under strictly anaerobic conditions at 37°C and pH 5 in chemically defined medium (without yeast extract) either on CO (60% CO and 40% Ar; AS Eesti AGA) or syngas (50% CO, 20% H_2_, 20% CO_2_, 10% Ar; AS Eesti AGA). Four independent experiments were conducted with cultures grown at dilution rates (*D*) of ~1.0 and ~2.0 day^−1^ on both feed gas mixes. Chemostat cultures were performed in 1.4-liter Multifors bioreactors (Infors AG) at a working volume of 750 mL and connected to a Hiden HPR-20-QIC mass spectrometer (Hiden Analytical) for online high-resolution off-gas analysis. The system was equipped with peristaltic pumps, mass flow controllers, and pH, oxidation-reduction potential, and temperature sensors. Cultures were sampled for RNA extraction and subsequent transcriptome analysis using RNA-seq after the optical density, gas uptake, and production rates had been stable for at least one working volume. Each sample represented a single biological replicate.

### Preparation of total RNA extracts.

Full details of culture sampling, RNA extraction, and purification are reported in previous work (13). Briefly, culture samples were pelleted by centrifugation (4,000 × *g* for 10 min at 4°C) and treated with RNAlater (catalog number 76106, Qiagen) overnight at 4°C, and pellets were stored at −80°C until RNA extraction. Thawed cell pellets were disrupted with glass beads in 800 μL of RLT buffer (catalog number 74104, Qiagen) containing 10 μL of β-mercaptoethanol by using the Precellys 24 instrument with liquid nitrogen cooling (Bertin Technologies) before extracting total RNA using the RNeasy minikit (catalog number 74104, Qiagen). Next, RNA extracts were depleted of DNA using off-column TURBO DNase (catalog number AM2239, Invitrogen) followed by purification using the RNA Clean and Concentrator kit (catalog number R1018, Zymo). We used the NanoDrop 1000 instrument (Thermo Fisher Scientific) for verifying efficiency of RNA purification. The high quality and integrity of the total RNA extracts were confirmed by RNA integrity numbers (RIN) above 8.2 with TapeStation 2200 equipment (Agilent Technologies). Total RNA and residual DNA concentrations were determined using the Qubit 2.0 instrument (Invitrogen).

### Removal of rRNA and RNA-seq library construction.

Total RNA extracts for each sample were split between the NuGEN, Qiagen, and Zymo kits for rRNA removal and strand-specific RNA-seq library construction according to vendor instructions. Samples referred to as “NuGEN” were processed with Universal Prokaryotic RNA-seq, prokaryotic AnyDeplete (catalog number 0363, NuGEN); “Qiagen” samples were processed with a QIAseq FastSelect 5S/16S/23S kit (catalog number 335925, Qiagen) (for rRNA removal) and a QIAseq stranded RNA Lib kit (catalog number 180743, Qiagen) (for library construction); “Zymo” samples were processed with a Zymo-Seq RiboFree total RNA library kit (catalog number R3000, Zymo Research).

### RNA sequencing and data analysis.

RNA sequencing of the 12 mRNA libraries (four cultures times three kits) was performed on a MiSeq instrument (Illumina) using the MiSeq v3 150 cycle sequencing kit (catalog number MS-102-3001, Illumina) with paired-end 2 × 75 bp reads. Raw RNA-seq data of the reference data set (high biomass concentration samples; GEO accession number GSE90792) (6) were analyzed together with the data generated in this work to ensure comparability. Full details of RNA-seq data analysis, including R scripts, were reported in previous work (13). Shortly, quality of sequencing reads was verified using MultiQC (20) and presence of read duplicates was examined using PicardTools (21). High-quality reads were then mapped to the NCBI reference genome of *C. autoethanogenum* NC_022592.1 (14) and genomic features were assigned using Rsubread (22). Strandedness of reads for the strand-specific data of NuGEN, Qiagen, and Zymo was calculated using RSeQC v3.0.1 (23). Finally, raw library sizes were normalized and transcript abundances were estimated as RPKM by using edgeR (24) (see Table S1 for RPKM data). rRNA transcript abundances were removed prior to data analysis for the results shown in Fig. 1 to avoid bias from varied efficiencies of rRNA removal between kits. Hierarchical clustering of individual transcript abundances (Fig. 1C) was performed using the ComplexHeatmap package in R (version 2.10.0) (25).

### Data availability.

The RNA-seq data have been deposited in the NCBI Gene Expression Omnibus repository under accession number GSE200959.
